# Factors associated with preventable infant death: a multiple logistic regression

**DOI:** 10.11606/S1518-8787.2018052000252

**Published:** 2018-04-20

**Authors:** Sandra Maria Cunha Vidal e Silva, Rogério Antonio Tuon, Livia Fernandes Probst, Brunna Verna Castro Gondinho, Antonio Carlos Pereira, Marcelo de Castro Meneghim, Karine Laura Cortellazzi, Glaucia Maria Bovi Ambrosano

**Affiliations:** I Prefeitura Municipal de Piracicaba. Secretaria Municipal de Saúde. Coordenação do Programa Pacto pela Redução do Óbito Infantil. Piracicaba, SP, Brasil; II Universidade Estadual de Campinas. Faculdade de Odontologia de Piracicaba. Programa de Pós-Graduação em Odontologia, Área de Concentração Saúde Coletiva. Piracicaba, SP, Brasil; III Universidade Estadual de Campinas. Faculdade de Odontologia de Piracicaba. Departamento de Odontologia Social. Piracicaba, SP, Brasil

**Keywords:** Infant Mortality, Risk Factors, Socioeconomic Factors, Perinatal Care, Infant, Newborn, Diseases, prevention & control, Prenatal Care, Cross-Sectional Studies

## Abstract

**OBJECTIVE:**

To identify and analyze factors associated with preventable child deaths.

**METHODS:**

This analytical cross-sectional study had preventable child mortality as dependent variable. From a population of 34,284 live births, we have selected a systematic sample of 4,402 children who did not die compared to 272 children who died from preventable causes during the period studied. The independent variables were analyzed in four hierarchical blocks: sociodemographic factors, the characteristics of the mother, prenatal and delivery care, and health conditions of the patient and neonatal care. We performed a descriptive statistical analysis and estimated multiple hierarchical logistic regression models.

**RESULTS:**

Approximatelly 35.3% of the deaths could have been prevented with the early diagnosis and treatment of diseases during pregnancy and 26.8% of them could have been prevented with better care conditions for pregnant women.

**CONCLUSIONS:**

The following characteristics of the mother are determinant for the higher mortality of children before the first year of life: living in neighborhoods with an average family income lower than four minimum wages, being aged ≤ 19 years, having one or more alive children, having a child with low APGAR level at the fifth minute of life, and having a child with low birth weight.

## INTRODUCTION

Infant mortality is a classic indicator of the socioeconomic conditions of a country or region^1^. Infant mortality rate is defined as the number of deaths of children under one year of age, per thousand live births, in the population living in a given geographical area, in the year under consideration. It consists of two components: the neonatal period, which estimates the risk of death in the first 27 days of life, and the post-neonatal period, which estimates the risk of death from 28 days to the end of the first year of life^2^.

While neonatal mortality is intrinsically related with the conditions of pregnancy, delivery, and the child's own physical integrity, post-neonatal mortality is more associated with socioeconomic and environmental conditions, with a predominance of infectious causes. In both components of infant mortality, however, an important share of responsibility is attributed to health services and social determinants of health. Adequate sanitary measures, access to health services, and good quality of care are factors that play a role in reducing infant mortality^3^.

The post-neonatal period showed greater decrease (50%) compared to the neonatal period (death in the first 27 days of life), in which a reduction of 36.5% was observed, from 2000 to 2006^2^. The prevalence of neonatal death still remains high, especially for the early neonatal component (up to six days), with the aggravating circumstance that most of these deaths are considered preventable^2^.

In Brazil, even with regional differences, important changes occurred in the health conditions of children in the last two decades. The infant mortality rate showed a significant reduction, with rates ranging from 21.1 (in 2000) to 15.3 (in 2011), corresponding to a decrease of 27.5%^4^. This was mainly due to the reduction in the mortality of children in the post-neonatal period. However, there was an increase in mortality in the neonatal period and markedly in the first week of life, that is, approximatelly 60% of the deaths occur in the early neonatal period^5^.

Therefore, the analysis of child mortality according to the basic cause of death is essential as it allows direct actions to be performed to avoid the initial causes of the process that leads to death^6^.

The study of preventable deaths is considered a methodological approach to assess the quality of health services, and its occurrence is considered an indicator of the potential shortcomings of the health system in the provision of the necessary care at the appropriate time^7^. High rates of avoidable infant deaths reflect difficulties in the access and quality of the health services offered to the population^8^.

Since the 1990s, approximately 60% of the infant deaths are concentrated in the first few days of life from complications in pregnancy, prenatal period, and delivery. These determinants associated with neonatal death reflect multifactorial influences such as the social status of the family, maternal characteristics, and access and quality of maternal and child care^9^.

The report of the National Commission on Social Determinants of Health highlights two aspects of inequities in health^10^ related with wealth distribution in the country and the deterioration in the relations of solidarity and trust among the population. Countries with high income inequalities have poor levels of social cohesion and political participation and invest less in social support networks, which are essential for the promotion and protection of health^10^.

Environmental conditions, the characteristics of the mother, and social relationships between population groups are also determining factors for the death of a child. Therefore, factors such as the age of the mother, interval between pregnancies, parity, low birth weight, sanitation, access to health centers, and other elements that are part of the life of a person such as housing, income, security, and education, are associated with child mortality^5^. Furthermore, the standard of living of a society is hard to be measured because of its complexity, since its social relationships encompass different behaviors and cultural values, as well as different political relations between persons and ethnic groups. These values depend on the time, place, and life course of each society^11^.

Therefore, the identification of pregnant women at higher risk, the reduction of organizational barriers in health services, and the increase of the access to family planning and early diagnosis of pregnancy are strategic actions to reduce the child mortality rate^12^. This research aimed to study preventable child mortality and its associated factors.

## METHODS

This study was approved by a local Research Ethics Committee, with resolution 196/96 of the National Health Council, Brazilian Ministry of Health, under Process 129/2012.

This is an analytical cross-sectional study with preventable child mortality as dependent variable. Data were obtained from the Information System on Live Births (SINASC) and Information System on Mortality (SIM)^13^ in the period from 2005 to 2011, in the city of Piracicaba, state of São Paulo, Brazil. Technicians of the Municipal Database performed the link between the data from SINASC and SIM. The fields used for linking were the data on the identification of the mother (full name, age, and address). Two files were made per year, in series from 2005 to 2011: one for preventable deaths (excluding other classifications) and another for live births, in which the death was marked in red. All the files of the variables of personal identification were removed, leaving only the variable of the neighborhood of residence of the mothers. Manual peer review was carried out by the technical officials of the municipality database.

The average income of the neighborhood of the mother was obtained from the 2010 IBGE Census^14^. The variable of attachment for live births was the neighborhood of the mother.

Child deaths considered non-preventable were excluded from this study according to the classification criteria adopted by the State System of Data Analysis Foundation (SEADE), as well as the ones whose mothers did not live in Piracicaba^15^.

To compose the sample, 272 preventable deaths were selected after the exclusion of 88 non-preventable ones in a total of 360 registered deaths in the SIM in the studied period. To study live births (control group), from a population of 34,284 babies (SINASC), a sample was chosen from a systematic sampling of 1/8 of this population, in which we obtained 50 births per month. A recorded number was drawn to start the selection, and from there, one case in every eight live births was systematically selected, composing a sample of 629 for each year studied, which amounted to 4,402 cases.

The sample size was determined assuming a 95% confidence level, sampling error of 5%, test power of at least 80%, and significance level of 5%. The independent variables were grouped into four hierarchical blocks according to the model proposed by Lima et al.^16^, organized in the levels: distal, intermediate I and II, and proximal in relation to the dependent variable. In this context, the distribution of the variables in each hierarchical block started with sociodemographic factors until comprising those with greater proximity to the outcome variable, as follows:

- Block I. Distal variables – sociodemographic characteristics of the mother: Education of the mother: none, from one to three years, from three to seven years, from eight to 11 years, 12 years or more. Profession of the mother: housewife, student, other. Race: white, black, yellow (Asian), mixed race. Average income in minimum wage in the neighborhood of the mother: up to 3.99 minimum wages, from four to 9.99 minimum wages, 10 or more minimum wages.
- Block II. Intermediate variables I –characteristics of the mother: Age group of the mother: from 10 to 14 years old, from 15 to 19 years old, from 20 to 29 years old, from 30 to 39 years old, 40 years old or more. Number of children alive: zero, from one to three, four or more. Number of dead children: zero, one, two or more. Marital status of the mother: single, married, widowed, divorced.
- Block III. Intermediate variables II – Prenatal and delivery care: Type of pregnancy: singleton, twins, triplets or more. Type of delivery: vaginal birth, cesarean section, other. Number of prenatal care appointments: none, from one to three, from four to seven, seven or more.
- Block IV. Proximal variables – Health conditions of the newborn and neonatal care: Pregnancy duration in weeks: less than 28 weeks, from 28 to 37 weeks, from 37 to 40 weeks, 40 or more weeks. APGAR score (which assesses the vitality of the newborn in the first, fifth, and tenth minute of life) in the first minute of life: from 0 to 3, from 4 to 7, from eight to 10. APGAR score in the fifth minute of life: from 0 to 3, from 4 to 7, from eight to 10. Birth weight (in g): less than 1,000 g, from 1,000 g to 1,499 g, from 1,500 g to 2,499 g, from 2,500 g to 3,999 g, 4,000 g or more.

Initially, descriptive analyses were performed, which were followed by the estimation of hierarchical multiple logistic regression models with the PROC GENMOD procedure using the SAS statistical program, considering the binomial distribution and logistic link function. Variables had as permanence condition the p ≤ 0.05 model and the settings were evaluated according to the Quasi-Likelihood under Independence Model Criterion (QIC). All analyses were carried out in the SAS statistical program^17^.

## RESULTS

Table 1 shows the frequency of preventable child deaths, considering the underlying causes of death and components of child mortality. Regarding the underlying causes of death, 272 deaths occurred in the studied period, of which 35.3% (n = 96) could have been avoided with adequate diagnosis and early treatment during pregnancy, 26.8% (n = 73), with the appropriate care of the pregnant woman, 22.1% (n = 60), with partnerships with other sectors (intersectoriality), 15.4% (n = 42), with the proper care of the woman during delivery, and 0.4% (one death), with immunoprevention. For components of child mortality, we observed that the higher frequency of deaths occurred in the early neonatal period (between zero to six days of life), with a total of 141 deaths (51.8%). In the late neonatal period (from seven to 27 days), there were 48 deaths (17.6%). The sum of child deaths that occurred from zero to 27 days (n = 189) corresponds to the component “neonatal child mortality”; approximately 69.4% of the deaths happened during this particular period. In the post-neonatal period (from 28 to 364 days), we observed a percentage of 30.5%, which corresponds to 83 deaths.

**Table 1:** Frequency distribution of variables related to children who died from potentially preventable causes. Piracicaba, state of São Paulo, Brazil, 2011.

Variable	Category	Frequency	%
Cause of death	Reducible by immunoprevention	1	0.4
	Reducible by adequate care for women during pregnancy	73	26.8
	Reducible by adequate care for women during labor	42	15.4
	Reducible by adequate actions of early diagnosis and treatment	96	35.3
	Reducible by partnerships with other sectors	60	22.1
Components of child mortality	Early neonatal death (0 to 6 days of life)	141	51.8
	Late neonatal death (from 7 to 27 days)	48	17.6
	Post-neonatal death (from 28 days to a year)	83	30.5

The frequency of variables regarding the sociodemographic aspects (distal level) and characteristics (intermediate level I) of the mother is shown in Table 2. At the distal level, we observed that the percentage of child mortality decreased the higher the educational level of the mother. Among student mothers, the percentage of child death was 6.1%, and among mothers who declared themselves as black, the percentage was 10.1%. We also found that mortality increased with decreasing average income of the neighborhood of the mother. At the intermediate level I, 10.3% of the children whose mothers were aged between 10 and 14 years ended up dying; this percentage dropped as the mothers got older, reaching a mortality of 4.4% in the age group from 30 to 39 years old. Regarding the number of children alive, the highest percentage of death (8.7%) was observed among mothers with four or more children alive. Among mothers who were pregnant for the first time (with zero children) and mothers with two or more dead children, the percentage of child death was 5.5% and 16.9%, respectively, while among mothers who had no dead children, the percentage was 5.2%.

**Table 2:** Frequency distribution of variables regarding the sociodemographic characteristics (distal level) and personal characteristics (intermediate level I) of the mother. Piracicaba, state of São Paulo, Brazil, 2011.

Variable	Category	Preventable child death			
		No		Yes	
		Frequency	%	Frequency	%
Block I – Distal level					
Education level of the mother	None	8	88.8	1	11.1
	1 to 3 years	180	90.9	18	9.0
	4 to 7 years	1,090	92.8	84	7.1
	8 to 11 years	2,583	95.2	128	4.7
	12 and more	541	94.9	29	5.0
Profession of the mother	Housewife	1,802	94.0	115	6.0
	Student	139	93.9	9	6.0
	Other paid jobs	2,080	94.6	118	5.3
Race	White	3,245	94.3	193	5.6
	Black	222	89.8	25	10.1
	Yellow (Asian)	6	100.0	0	0.0
	Mixed race	876	95.2	44	4.7
Average income of neighborhood in minimum wages	Zero to 3.99	1,486	92.9	112	7.0
	4 to 10	2,559	94.9	136	5.0
	10 or more	298	95.8	13	4.1
Block II – Intermediate level I					
Ageof the mother	10 to 14 years	26	89.7	3	10.3
	15 to 19 years	617	92.1	53	7.9
	20 to 29 years	2,355	94.3	143	5.7
	30 to 39 years	1,328	95.6	61	4.4
	40 or more	76	89.4	9	10.6
Number of alive children	Zero	2,086	94.4	123	5.5
	1 to 3	2,146	94.4	126	5.5
	4 or more	166	91.2	16	8.7
Number of dead children	Zero	4,100	94.7	228	5.2
	One	247	91.4	23	8.5
	2 or more	49	83.0	10	16.9
Marital status	Single	1,524	93.5	106	6.5
	Married	2,791	95.8	124	4.2
	Widow	5	71.4	2	28.6
	Divorced	80	93.0	6	7.0

Table 3 presents the frequency of variables regarding prenatal and delivery care (intermediate level II) and the health conditions of the newborn and neonatal care (proximal level). Among the women pregnant with twins, the percentage of child death was 21.9%, while among the ones with singleton pregnancy, the percentage was only 5.2%. In vaginal birth, 6.7% of the children ended up dying, and in cesarean section, 4.7%. The percentage of child death decreased as the number of medical appointments increased. At the proximal level, we observed that among children who were born in less than 28 weeks of gestational age, the percentage of child death was 81.6%; those with gestational age between 28 and 36 weeks had a percentage of 60.4%; those with gestational age between 37 and 41 weeks had a percentage of 12.6%, and those with gestational age of 42 or more weeks, had a percentage of 2.5%. For the group of children with APGAR score from zero to three in the first and fifth minute, the death percentage was 69.2% and 78.2%, respectively. Among children with birth weight below 1,000 g, the percentage of death was 86.3%, and among those who were born weighing between 1,000 g and 1,500 g, this value decreased to 56.9%. For children who were born weighing between 2,500 g and 4,000 g, death percentage was only 2.6%.

**Table 3:** Frequency distribution of variables related to prenatal care and childbirth (intermediate level II) and the health of the newborn and neonatal care (proximal level). Piracicaba, state of São Paulo, Brazil, 2011.

Variable (Intermediate level II)	Category	Preventable child death			
		No		Yes	
		Frequency	%	Frequency	%
Block III – Intermediate level II					
Type of pregnancy	Singleton	4,303	94.7	239	5.2
	Twins	96	78.0	27	21.9
	Triplets or more	3	100.0	0	0
Type of delivery	Vaginal	1,528	93.2	110	6.7
	Caesarean section	2,874	95.2	142	4.7
	Other	0	0.0	15	100.0
Number of prenatal visits	None	26	76.4	8	23.5
	1 to 3	147	80.7	35	19.2
	4 to 6	742	90.2	80	9.7
	7 or more	3,484	96.9	109	3.0
Block IV – Proximal level					
Duration of pregnancy in weeks	28 to 36	32	39.5	49	60.4
	37 to 41	270	87.3	39	12.6
	42 and more	4,081	97.4	108	2.5
APGAR score in the first minute	0 to 3	36	30.7	81	69.2
	4 to 7	332	85.5	56	14.4
	8 to 10	3,824	97.8	85	2.1
APGAR score in the fifth minute	0 to 3	10	21.7	36	78.2
	4 to 7	64	52.4	58	47.5
	8 to 10	4,120	96.9	128	3.0
Weight of the child at birth (g)	Less than 1,000	13	13.6	82	86.3
	1,000 I— 1,499	25	43.1	33	56.9
	1,500 I— 2,500	320	87.4	46	12.5
	2,500 I— 4,000	3,843	97.3	104	2.6
	4,000 or more	201	99.0	2	0.9

Table 4 presents the estimated parameters of multiple hierarchical logistic regression models adjusted to describe the influence of the variables on the death of newborns in hierarchical levels. In model 1 (distal level), comprising the sociodemographic characteristics of the mother, only the variable “average income per family of the neighborhood of the mother” (in minimum wages) remained in the multiple regression model (p < 0.0001). In model 2, which included the intermediate level I (the characteristics of the mother), the following variables remained: average income of the neighborhood of the mother (p < 0.0001), age group of the mother ≤ 19 years (p < 0.0001), mothers with four or more children (p = 0.0001), mothers with one dead child (p = 0.0219), and mothers with two or more dead children (p = 0.0017). In model 3, which included the intermediate level II and the studied variables related to prenatal care and delivery, the variables that remained were those concerning the average income of the neighborhood of the mother (p < 0.0001), the age of the mother ≤ 19 years (p < 0.0001), mothers with four or more living children (p = 0.0001), mothers with one dead child (p = 0.0260), mothers with two or more dead children (p = 0.0017), and mothers pregnant with more than one child (p < 0.0001). After adjusting the four hierarchical levels, the variables that were part of the final model were: a) distal level (block I): average income of the neighborhood of the mother (p < 0.0001); b) intermediate level I (block II): mothers aged ≤ 19 years (p < 0.0001), mothers with one to three alive children (p = 0.0028), mothers with four or more alive children (p < 0.0001), and mothers with two or more dead children (p = 0.0249); and, c) proximal level (block IV): newborns with low APGAR score (p < 0.0001) and low birth weight (p < 0.0001). From the analysis of the hierarchical multiple logistic regression, we can state that children whose mothers were aged ≤ 19 years (p < 0.0001), lived in neighborhoods with average income of less than 4 minimum wages (p < 0.0001) per family, (p < 0.0001), had one to three alive children (p = 0028), had four or more alive children (p < 0.0001), had two or more dead children (p = 0.0249), had children whose APGAR score in the fifth minute was low (p < 0.0001), and had children with low birth weight (p < 0.0001) had higher chances of preventable child mortality.

**Table 4:** Estimated parameters of hierarchical multiple logistic regression models adjusted to describe the influence of the variables on child deaths. Piracicaba, state of São Paulo, Brazil. 2011.

Variable		Model 1			Model 2			Model 3			Model 4 (Final model)		
		Estimate	SE^a^	p	Estimate	SE	p	Estimate	SE	p	Estimate	SE	p
Intercept		-1.91	0.01	< 0.0001	-2.38	0.09	< 0.0001	-2.49	0.10	< 0.0001	7.25	0.65	< 0.0001
Distal level													
Average household income in the neighborhood													
	Zero to 3.99	Ref^b^			Ref			Ref			Ref		
	4 to 10	-086	0.09	< 0.0001	-0.61	0.10	< 0.0001	-0.64	0.09	< 0.0001	-0.51	0.13	< 0.0001
	10 or more	-1.20	0.23	< 0.0001	-0.88	0.23	0.0001	-0.87	0.24	0.0003	-0.98	0.33	0.0030
Intermediate level I													
Age group													
	≤ 19 years old				0.61	0.10	< 0.0001	0.68	0.11	< 0.0001	0.60	0.14	< 0.0001
	20 to 39 years old (ref)				Ref			Ref			Ref		
	< 40 years old				0.48	0.30	0.1081	0.52	0.31	0.0924	0.40	0.31	0.1968
Alive children													
	Zero (ref)				Ref						Ref		
	1 to 3				0.11	0.10	0.2364	0.13	0.10	0.1797	0.34	0.11	0.0028
	4 or more				0.68	0.22	0.0016	0.72	0.20	0.0003	1.05	0.21	< 0.0001
Dead children													
	Zero (ref)				Ref						Ref		
	One				0.35	0.16	0.0273	0.34	0.16	0.0330	0.31	0.20	0.1116
	2 or more				0.99	0.31	0.0012	1.00	0.32	0.0015	0.64	0.29	0.0251
Intermediate level II													
Pregnancy													
	Singleton (ref)							Ref					
	Twins or more							1.33	0.21	< 0.0001			
Proximal level													
APGAR score in the fifth minute													
	0 to 3										Ref		
	4 to 7										-1.24	0.53	0.0190
	8 to 10										-3.13	0.49	< 0.0001
Weight of the child at birth (g)													
	Less than 1,000										Ref		
	1,000 I— 1,499										-1.28	0.47	0.0065
	1,500 I— 2,500										-2.66	0.35	< 0.0001
	2,500 I— 4,000										-4.04	0.34	< 0.0001
	4,000 or more										-6.10	0.55	< 0.0001
Adjustment of the model (QIC)^c^		2,123.95			2,030.19			1,988.99			1,128.06		

Ref: reference

a SE: Standard error of estimate.

b Ref: reference level. The reference level of the dependent variable (preventable child death) was the category “yes”.

c QIC Statistics: quasi-likelihood under the independence model criterion.

## DISCUSSION

In pregnant women care, aspects concerning the early detection of pregnancy, the access to health services, and mother and child care need to be considered^12^. The work with partnerships established by horizontal care for pregnant women and children, outpatient care (e.g., child health days, facility care, and emergency transport), equipped hospitals, and prepared teams would help reducing child mortality^18^ ^,^ ^19^. Our study showed that 73 children (26.8%) would not have died if their mothers had received better care during pregnancy, 60 (22.1%) would not have died if they had the help of (intersectoral) partnerships, 42 (15.4%) if they had appropriate care at delivery, and one (0.4%) if there was the chance to receive immunoprevention.

Regarding the underlying causes of preventable deaths, this study showed that 96 (35.3%) children from 272 deaths may not have died if, during prenatal care, mothers had had the chance of being diagnosed and treated for possible complications at the right time, and the main causes of death were related with maternal conditions that occurred during pregnancy, such as hypertension, urinary tract infection, vaginosis, gestational diabetes, or previous diabetes, corroborating with other scientific studies^12^ ^,^ ^20^. The impact of actions to end preventable infant deaths is high and the cost is low^19^.

Our study showed that, before the first year of life, children will have more chances of dying if their mothers fit at least one of the following criteria: live in neighborhoods with an average family income of < 4 minimum wages, aged ≤ 19 years, have one or more alive children, and have two or more dead children. When it comes to the children's own characteristics, newborns will have more chances of dying if they are born with low APGAR score at the fifth minute and low birth weight (< 2,500 g).

In the city of Piracicaba, even with the expansion of the Health Family Program and the greater technological adequacy of health services in secondary levels, the neonatal death rate still remains high, accounting for 69.5% of all child deaths (51.8% having died before six days of age), according to several studies in the literature^1^ ^,^ ^20^ ^-^ ^22^. Concerning the post-neonatal component, the proportion of deaths was 30.5%, and the main causes of death could also be prevented with early diagnosis and treatment. These causes are associated with respiratory disorders, epileptic syndrome, and infectious diseases, especially the ones acquired in the hospital environment when the period of hospitalization is extended^23^.

The literature discusses the causes of preventable death according to age group, sex, socioeconomic factors, the analysis of the impact of different prevention levels, and its reduction among different locations or ethnic groups^1^. In this study, we used the model of hierarchical multilevel analysis, analyzing its independent variables and composing hierarchical levels from distal to proximal events regarding child mortality. Higher risk factors of preventable child mortality are part of chains of cause and effect, in which only one chain could be enough for the occurrence of child death^12^ ^,^ ^24^.

In the block of sociodemographic factors, the variable “mothers who live in neighborhoods with average income of less than 4 minimum wages per family” was the only one associated with death that remained in the final model. The association between child death and low income has been discussed in the literature, showing that a country can overcome social inequalities with policies that promote a better distribution of wealth^8^ ^,^ ^10^. Thus, preventable causes of death with high rates of risk represent a problem in less fortunate groups and there is an ethical requirement to obtain evaluation criteria to solve this problem^10^ ^,^ ^24^ ^,^ ^25^. The socioeconomic condition of the mother has been considered as the factor of highest positive effect in reducing child mortality, followed by factors regarding care and demographic transition, all part of maternal characteristics^2^ ^,^ ^25^.

In this study, the children of teenage mothers, aged ≤ 19 years, have higher chances of preventable child death. Teen pregnancy concentrates the worst damage to the health of the mother and perinatal complications, which can worsen in precarious socioeconomic and geographical situations, with difficulties in the access to health services and with the presence of family issues^23^. Women of low socioeconomic level get pregnant early and have difficulties while taking care of newborns, since poverty and teen pregnancy are indicators of precarious life conditions and lack of prenatal care^26^.

Another study has shown that the lower the age of the mother, the greater are the death trends in the first year of life of the children, especially concerning postnatal deaths, associated with newborn care^26^. Therefore, teenagers need to be taken care of differently, with a multidisciplinary team to ensure that multifactorial demands are understood. The support to families and teenagers with an intersectoral network of health care becomes essential for the adequate care of mothers and children^26^.

In this study, mothers with one or more alive children had higher chances of preventable child death, corroborating another study that has reported higher prevalence of child death among mothers with four or more children^23^. In addition, mothers with two or more dead children were also significantly associated with preventable death. Abortions and early fetal deaths have been associated with a higher prevalence of child deaths^22^ ^,^ ^25^.

In the final model, low APGAR scores in the fifth minute and low birth weight showed association with preventable child death, corroborating studies found in the literature and supportting its continued usefulness in contemporary practice^20^ ^,^ ^27^ ^–^ ^29^. Premature newborns have a ten times higher chance of death because of complications regarding longer permanence in the hospital or the undevelopment of the neurological and respiratory systems^22^ ^,^ ^25^ ^,^ ^30^.

In this context, the risk factors highlighted in this study indicate the need for actions that improve the planning of prenatal care by detecting and treating pregnancy difficulties at the right moment and taking care of women since the beginning of gestation with the help of all offered services, in addition to, the development of horizontalized health care networks, the broadening of points of view on social inequalities, the planning of different actions for teenage groups, multiparous women, and women with previous dead children, and the development of actions that omprove the prenatal care, which can contribute to reduce preventable child deaths.

Finnaly, our study has some limitations. This was a cross-sectional study that sought inferences regarding causal factors without, however, establishing a temporal relationship. Furthemore, data was manually collected by specialized and trained technicians. However, despite the process of conferencing, the human factor can be considered a limitation of the study.

In conclusion, the following characteristics of the mother are determinant for the higher mortality of children before the first year of life: living in neighborhoods with an average family income lower than four minimum wages, being aged ≤ 19 years, having one or more alive children, having children with low APGAR level at the fifth minute of life, and having low birth weight (< 2,500 g).
